# SIRT1 rs7069102 Polymorphism Confers Increased Risk of Diabetic Retinopathy in T2DM

**DOI:** 10.3390/genes17020221

**Published:** 2026-02-10

**Authors:** Melina Bešić, Jernej Letonja, Mojca Globočnik Petrovič, Ana Peterlin, Ema Šuligoj, Stella Stare, Daniel Petrovič

**Affiliations:** 1 Laboratory for Histology and Genetics of Atherosclerosis and Microvascular Diseases, Faculty of Medicine, University of Ljubljana, Korytkova 2, 1000 Ljubljana, Sloveniaana.peterlin@mf.uni-lj.si (A.P.);; 2 Institute of Histology and Embryology, Faculty of Medicine, University of Ljubljana, Korytkova 2, 1000 Ljubljana, Slovenia; 3 Eye Hospital, University Medical Centre Ljubljana, 1000 Ljubljana, Slovenia; 4 Faculty of Medicine, University of Ljubljana, Korytkova 2, 1000 Ljubljana, Slovenia

**Keywords:** diabetic retinopathy, T2DM, SIRT1, rs7069102, TNF-α, rs1800629

## Abstract

Background: The incidence and prevalence of type 2 diabetes mellitus (T2DM) has been increasing worldwide recently. Diabetic retinopathy (DR) is a major ocular complication of diabetes mellitus, and it is the leading cause of blindness and visual impairment. Sirtuin 1 (SIRT 1) is a NAD+-dependent deacetylase and is involved in stress responses such as hypoxic and genotoxic stress, inflammation and heat shock. Tumor necrosis factor α (TNF-α) is an important inflammatory mediator that is involved in the pathogenesis of T2DM. The purpose of our study was to investigate the relationship between the *SIRT1* rs7069102 polymorphism and *TNF*- α rs1800629 polymorphisms and diabetic retinopathy (DR) in patients with type 2 diabetes mellitus (T2DM). Materials and Methods: We analyzed 1554 Slovenian (Caucasian) patients with T2DM of at least 10 years’ duration, stratifying them into two groups: 577 patients with diabetic retinopathy (DR) and 977 patients without DR. Genotyping of SIRT1 rs7069102 and TNF-α rs1800629 polymorphisms was performed using the StepOne real-time PCR System with TaqMan SNP Genotyping Assays. Results and Conclusions: A significant difference in the distribution of *SIRT1* rs7069102 genotypes and alleles was observed between the groups. Under the dominant inheritance model, patients with CC or CG genotypes were more likely to develop DR than those with the GG genotype (OR = 1.30; 95% CI = 1.02–1.65; *p* = 0.036). No significant association was found between *TNF-α* rs1800629 and DR.

## 1. Introduction

The incidence and prevalence of type 2 diabetes mellitus (T2DM) is increasing worldwide [1]. As the major ocular complication of diabetes mellitus, diabetic retinopathy (DR) is among the leading causes of blindness and visual impairment [2]. It is a complex condition influenced by multiple factors, with various structural, biochemical, molecular, and functional abnormalities playing a role in its development [3,4]. DR affects more than 103.12 million people and is the most common microvascular complication in people with diabetes and can cause incorrigible vison loss [5]. The number of patients with DR is expected to increase to 643 million by 2030 and 783 million by 2045 globally [6]. According to epidemiological projections for 2030, the prevalence of diabetic retinopathy (DR) is expected to increase at a relatively low rate in traditionally high-income regions, such as North America and Europe, with an increase ranging from 10.8% to 18.0%. In contrast, the rates of increase in middle- and low-income regions, including the Western Pacific, South and Central America, Asia, Africa, and the Middle East and North Africa (MENA), are considerably higher, with increases ranging from 20.6% to as much as 47.2% [7]. Key risk factors for the development and progression of DR include hypertension, the duration of diabetes, poor glycemic and lipid control, and genetic predisposition [8,9]. Constant hyperinsulinemia, hyperglycemia and oxidative stress result in microvascular complications (nephropathy, neuropathy and retinopathy) and macrovascular complications of diabetes [10]. Genetic factors play a major role in both T2DM and DR. Genetic factors contribute to approximately 25–50% of the risk for developing DR [11,12]. The most important genes associated with diabetic retinopathy are located on chromosomes 1–22, except for chromosomes 5, 8, 11, 12, 14, 18, 21, and X/Y. The largest number of genes related to the development of retinopathy is found on chromosome 1 (*SELP*, *MTHFR*, *NVL*, and *CRP*) and 7 (*IL-6*, *eNOS*, *AR*, and *PAI-1*). Studies on the relationship between the development of retinopathy and polymorphisms are focused on the *VEGF* gene (chromosome 6), *ACE* (chromosome 17), and *APOE* (chromosome 19) [13].

Sirtuin 1 (SIRT1) is an NAD^+^-dependent deacetylase belonging to the silent information regulator family of enzymes and is expressed in multiple tissues, including adipose tissue, muscle, the pancreas, the liver, and the kidney [14]. Through histone deacetylation, SIRT1 may confer antidiabetic effects by influencing insulin secretion and attenuating insulin resistance via regulatory actions on insulin signaling pathways, inflammation, mitochondrial function, and circadian rhythms [14]. SIRT1 regulates a range of cellular processes, including stress responses (hypoxic and genotoxic stress, inflammation, and heat shock), as well as glucose and lipid metabolism in patients with T2DM. It also enhances insulin secretion in the pancreatic beta cells, increases glucose uptake in skeletal muscle, mobilizes lipids in adipose tissue, induces mitochondrial biogenesis, and enhances insulin sensitivity [15]. Jaliffa et al. showed in their study with mice that SIRT1 is expressed in the mouse cornea, lens, iris, ciliary body, inner nuclear layer, outer nuclear layer, and retinal ganglion cell layer [16]. Müller cells, which are specialized glial cells, are involved in the pathogenesis of retinopathy and are considered a principal contributor to retinal inflammation [17,18]. Müller cells produce pro-inflammatory cytokines to restore retinal homeostasis in diabetes [19]. SIRT1 functions as a sensor of cellular energy status, mediating the connection between metabolic stress and adaptive cellular mechanisms [20,21].

In diabetes, the expression of SIRT1 is reduced in both the retina and its vasculature [22]. The activation of endogenous SIRT1 in the ischemic retina is essential under stressful conditions to safeguard against retinopathy.

Tumor necrosis factor α (TNF-α) is an important inflammatory mediator that is involved in the pathogenesis of T2DM [23]. It regulates inflammation through the production of reactive oxygen species (ROS), as well as through the regulation of transcription. TNF-α also induces the disruption of tight junctions between the endothelial cells in the retina, stimulates neovascularization, stimulates leukocyte adhesion through the upregulation of ICAM-1 and VCAM-1 and induces the cell death of pericytes and endothelial cells [24]. Higher vitreal and serum levels of TNF-α indicate a higher degree of retinal involvement in patients with diabetes [25,26]. SIRT1 and TNF-α regulate each other in physiological and DR conditions. SIRT1 suppresses the production of TNF-α and other pro-inflammatory cytokines. However, in diabetes, SIRT1 is downregulated by hyperglycemia-induced high levels of TNF-α through epigenetic silencing via miR-34a and miR-200b, as well as a reduction in its activity due to the depletion of NAD+. The anti-TNF-α effect is the main protective effect of SIRT1 in DR [27,28].

In this study, we examined the associations between the SIRT1 rs7069102 and TNF-α rs1800629 polymorphisms and diabetic retinopathy in Slovenian patients with T2DM.

## 2. Materials and Methods

### 2.1. Patients

This retrospective association study included 1554 Slovenian patients with T2DM of more than 10 years’ duration, who were divided into two groups based on the presence or absence of retinal pathology. The first group included 577 patients with DR, whereas the second group comprised 977 patients without DR. Patients with advanced nephropathy or ocular diseases of other etiologies were excluded from this study. We collected patient data through a questionnaire, including age, sex, T2DM duration, systolic and diastolic blood pressure, duration of arterial hypertension, smoking status, diabetic retinopathy and its duration, other microvascular complications, and coronary artery disease. The fundus was examined using slit lamp biomicroscopy with a non-contact lens by a senior ophthalmologist (M.G.P.) after pupil dilatation with tropicamide and phenylephrine. The results were documented electronically, employing a fundus camera with a 50°-angle (Topcon-TRC 40-IX; Tokyo, Japan). DR was classified in accordance with the criteria established by the Early Treatment Diabetic Retinopathy Study (ETDRS). We measured the participants’ height, weight, and waist circumference. Peripheral venous blood samples were drawn from the participants’ cubital vein, and the blood was used for biochemical and genetic analysis.

All participants signed informed consent for participation in this study. This study was conducted in accordance with the Declaration of Helsinki and approved by the Slovenian Commission for Medical Ethics. The ethics approval number for our study was 0120-105/2025.

### 2.2. Biochemical Analyses

Levels of HbA1c, total cholesterol, HDL, LDL, and triglycerides were assessed using established biochemical methods.

### 2.3. Genotyping

Genotyping was carried out according to the laboratory protocol described in our earlier publication [29].

### 2.4. Statistical Analysis

Allele discrimination plots were generated using StepOne Software version 2.2 (Applied Biosystems, Foster City, CA, USA). Statistical analyses were performed using IBM SPSS Statistics for Windows version 26.0 (IBM Corp., Armonk, NY, USA). Continuous variables were compared with Student’s t-tests or Mann–Whitney U tests, whereas categorical variables were compared using Chi-square tests. Logistic regression analysis was employed to examine the association between rs7069102 and rs1800629 polymorphisms and DR, after adjusting for confounding variables. A *p* value of less than 0.05 was considered statistically significant. Fisher’s exact test was used to assess potential deviation from Hardy–Weinberg equilibrium (HWE) (http://ihg.gsf.de/, accessed on 25 November 2025). The power of our study was 0.84.

## 3. Results

The clinical and laboratory profiles of the DR and non-DR patient groups are detailed in Table 1. Statistically significant differences between the two groups were observed for the following parameters: waist circumference (*p* < 0.001), BMI (*p* = 0.041), fasting glucose (*p* = 0.0015), S-HbA1c (*p* < 0.001), T2DM duration (*p* < 0.001), the presence of CAD (*p* < 0.001) and CAD duration (*p* < 0.001), diabetic neuropathy (*p* < 0.001) and insulin therapy (*p* < 0.001). The groups did not differ significantly with respect to age, sex, systolic or diastolic blood pressure (SBP and DBP, respectively), total cholesterol, HDL- or LDL-cholesterol, triglycerides, duration of arterial hypertension, or active smoking. In contrast, patients with DR showed increased waist circumference, higher BMI, longer T2DM duration, elevated fasting glucose and HbA1c, and a greater frequency of diabetic neuropathy. Genotype frequencies of investigated polymorphisms were in accordance with those predicted by the Hardy–Weinberg equilibrium (*p* > 0.05) in both groups.

Genotype and allelic distribution of the SIRT1 rs7069102 polymorphism of the 577 DR patients and 977 T2DM patients without DR are shown in Table 2. A statistically significant difference in genotype distribution was observed between the groups. The CG genotype was more prevalent in the group with DR than in the group without DR (*p* = 0.0343). After adjusting for BMI, waist circumference, fasting glucose, T2DM duration, HbA1c (%), CAD, diabetic neuropathy, and insulin therapy, the difference remained statistically significant (*p* = 0.029) (Table 2).

To evaluate the independent association of rs7069102 with DR, logistic regression analysis was conducted with adjustment for BMI, waist circumference, fasting glucose, T2DM duration, HbA1c (%), CAD, diabetic neuropathy, and insulin therapy (Table 3). Under the dominant inheritance model, patients with CC or CG genotypes had a higher likelihood of developing DR compared with those carrying the GG genotype (OR = 1.30; 95% CI = 1.02–1.65; *p* = 0.036). No statistically significant association between the CC genotype and DR was observed. According to the recessive model, the SIRT1 rs7069102 genotypes were not found to be associated with the risk of DR.

The genotype and allele distributions of the TNF-α rs1800629 polymorphism, along with the results of logistic regression, are presented in Table 4. No statistically significant differences were observed between the groups in either genotype or allele frequencies.

Additional statistical analysis using the Kruskal–Wallis test did not show an association between *SIRT1* rs7069102 genotypes and waist circumference, fasting glucose levels or HbA1c in either group (Table 5).

## 4. Discussion

Our findings indicate an association between the C allele of the SIRT1 rs7069102 polymorphism and DR in Slovenian patients with T2DM. Linear regression analysis, adjusted for confounding variables, revealed that individuals with the CG genotype were 1.33 times more likely to develop DR than those with the GG genotype (*p* = 0.029). Furthermore, under the dominant model of inheritance, participants with CC or CG genotypes had a 1.30-fold increased risk of DR compared with GG genotype carriers (*p* = 0.036). The recessive model of inheritance showed no significant correlation between the CC genotype and DR compared with CG and GG genotypes. On the other hand, we found no association between the TNF-α rs1800629 polymorphism and DR in either model of inheritance.

Currently, there are no published studies assessing the association between the SIRT1 rs7069102 polymorphism and DR. Our group found an association between rs7069102 and diabetic nephropathy in a Slovenian T2DM cohort of patients [29]. The presence of diabetic nephropathy was more frequent among individuals with the CC genotype compared to those carrying the CG or GG genotypes. Together, these studies support the CC genotype of the rs7069102 polymorphism as a promising genetic marker associated with increased risk of diabetic complications, at least within the Slovenian cohort.

However, Peeters et al. reported that the C allele of rs7069102 is associated with a reduced risk of visceral obesity in Belgian Caucasians [30]. They compared 1068 obese patients without diabetes or impaired glucose tolerance with 313 healthy controls. One possible interpretation of these findings is that the C allele may increase the risk of complications in patients with diabetes, and potentially confers benefits in a healthy population. However, the differences between the population of Belgian Caucasians and Slovenian Caucasians could also explain the difference in results.

Other SIRT1 polymorphisms were found to be associated with a susceptibility to T2DM development in an Iranian population [31] and T1DM in a Han Chinese population [32]. Dardano et al. reported that a different polymorphism of SIRT1 rs7896005 was associated with cardiovascular complications in Caucasian patients with T2DM in their 13-year-long observational study [33].

The expression of SIRT1 in diabetes and DR is a topic of several studies. Kowluru et al. reported a decreased expression of SIRT1 in human retinal samples from patients with DR, as well as in animal models. Hyperglycemia-driven oxidative stress downregulates SIRT1 which increases the activation of matrix metalloproteinase 9 (MMP-9) that promotes the development of DR [22]. Zeng et al. also reported decreased expression of SIRT1 and SIRT3 in their research on diabetic rats [34]. Similarly, Mishra et al. demonstrated, in their research on mice, that SIRT1 plays a crucial role in the development of diabetic retinopathy, as well as in the associated damage to retinal vasculature and neurons. Diabetic mice that overexpressed SIRT1 had comparable capillary density to non-diabetic mice; however, in regular diabetic mice it was significantly decreased [27]. To date, only one study has investigated the effect of the rs7069102 polymorphism on SIRT1 expression. Kilic et al. found that participants with CAD carrying the GG or CG genotypes had elevated plasma SIRT1 levels relative to those with the CC genotype; however, this association was not present in the control group [35].

Similarly to our findings, Kaidonis et al. did not report an association between the TNF-α rs1800629 polymorphism in patients with T2DM and sight-threatening DR, PDR or macular oedema in their study on a Caucasian UK population [36]. They did report an association between the A allele and risk for developing T1DM. Lindholm et al. reported similar results, but they found an association between the A allele and macrovascular complications in patients with T2DM [37]. Studies conducted on Japanese [38] and Chinese [39] populations also yielded similar results.

However, a positive association between the A allele of the rs1800629 polymorphism and PDR in Brazilian Caucasian patients with T2DM was reported by Sesti et al. [40]. In a recent study, Sanches-Valencia et al. found a positive association between the A allele of the rs1800629 polymorphism and DR in a Mexican population, yet the sample was restricted to only 203 diabetic patients [41].

Rs1800629 is also associated with the expression of TNF-α in patients with DN. Umapathy et al. reported that individuals with the AA genotype of rs1800629 exhibited higher circulating TNF-α levels compared with those carrying the GG genotype. This polymorphism is located in the promoter region of the TNF-α gene, which may account for the observed differences. Further research is required to confirm this hypothesis [42].

Interestingly, Wu et al. concluded that the rs1800629 polymorphism was associated with the risk of developing DN in a recent meta-analysis [43]. The A allele of the rs1800629 polymorphism was associated with an increased risk of developing T2DM in a Chinese Han population [44].

This cross-sectional case–control study is subject to certain limitations. The population investigated was relatively small and ethnically uniform, consisting of Slovenian patients with T2DM, both with and without DR. Another limitation is that we did not measure the circulating levels of SIRT1 and TNF-α. This is an important point for future research, as such information could be used to compare the cases and controls and investigate a possible association between the studied polymorphisms and the levels of SIRT1 and TNF-α. Other polymorphisms within the SIRT1 and TNF-α genes that may have contributed to the findings were also not investigated. An additional limitation of this study is its retrospective design. We are aware that some of the participants who, at the time of data collection, did not have a diagnosis of DR might develop DR in the future. A follow-up study in 10 years, for example, would address this limitation and is something our team will keep in mind. Also, the HWE of SIRT1 rs7069102 allele distribution in the group of patients with T2DM without DR was borderline (*p* = 0.0625); however, it is unlikely that it reflects a genotyping error or population stratification.

In conclusion, our study identified a significant association between the SIRT1 rs7069102 polymorphism and DR in T2DM, suggesting that this polymorphism may serve as a marker of susceptibility to DR in these patients. No association was observed between the TNF-α rs1800629 polymorphism and DR. The molecular mechanisms involved in DR are intricate, and a deeper understanding of these interactions could uncover new therapeutic targets for its treatment.

## Figures and Tables

**Table 1:** Clinical and laboratory characteristics of patients with (DR) (cases) and without DR.

	Case (N = 577)	Patients Without DR (N = 977)	*p Value*
**Age (years)**	65.3 ± 8.8	64.7 ± 9.6	*0.24*
**BMI (kg/m^2^)**	29.3 ± 4.5	29.7 ± 4.2	***0.041***
**Waist circumference (cm)**	107.4 ± 11.1	105.0 ± 11.3	***<0.001***
**SBP (mm Hg)**	150.0 [130.0–160.0]	150.0 [130.0–160.0]	*0.30*
**DBP (mm Hg)**	80.0 [80.0–90.0]	82.0 [77.2–90.0]	*0.19*
**Fasting glucose (mmol/L)**	8.7 ± 2.6	8.3 ± 2.2	***0.0015***
**Total cholesterol (mmol/L)**	4.7 [4.0–5.6]	4.7 [4.0–5.7]	*0.98*
**HDL-cholesterol (mmol/L)**	1.1 [1.0–1.3]	1.1 [1.0–1.4]	*0.074*
**LDL-cholesterol (mmol/L)**	2.7 [2.1–3.5]	2.7 [2.1–3.4]	*0.87*
**TGS-cholesterol (mmol/L)**	1.7 [1.2–2.3]	1.7 [1.2–2.4]	*0.36*
**HbA1c (%)**	7.93 ± 1.32	7.54 ± 1.10	***<0.001***
**T2DM duration (years)**	18.0 [15.0–25.0]	14.0 [12.0–17.0]	***<0.001***
**DR duration (years)**	5.0 [4.0–6.0]	0 [0.0–0.0]	
**AH duration (years)**	11.0 [6.0–18.0]	10.0 [6.0–15.0]	*0.063*
**CAD duration (years)**	8.0 [5.0–10.0]	8.0 [5.0–13.0]	***<0.001***
**Gender**			*0.21*
Male	300 (52.0%)	540 (55.3%)	
Female	277 (48.0%)	437 (44.7%)	
**Smoking (%)**			*0.088*
Yes (active)	54 (9.4%)	119 (12.2%)	
No (never + former)	523 (90.6%)	858 (87.8%)	
**AH**			*0.70*
Yes	474 (82.1%)	795 (81.4%)	
No	103 (17.9%)	182 (18.6%)	
**CAD**			***<0.001***
Yes	118 (20.5%)	352 (36.0%)	
No	459 (79.5%)	625 (64.0%)	
**Diabetic neuropathy**			***<0.001***
Yes	112 (19.4%)	96 (9.8%)	
No	465 (80.6%)	881 (90.2%)	
**Insulin therapy**			***<0.001***
Yes	432 (74.9%)	386 (39.5%)	
No	145 (25.1%)	591 (60.5%)	

Statistically significant values are written in bold. Abbreviations: BMI—body mass index; SBP—systolic blood pressure; DBP—diastolic blood pressure; HDL—high-density lipoprotein; LDL—low-density lipoprotein; TGS—triglycerides; HbA1c—glycated hemoglobin; T2DM—type 2 diabetes mellitus; DR—diabetic retinopathy; AH–arterial hypertension; CAD—cardiovascular diseases.

**Table 2:** Distribution of genotypes and alleles of the SIRT1 rs7069102 polymorphism in cases and patients without DR.

SIRT1 rs7069102	Case (N = 577)	Patients Without DR (N = 977)	*p Value*	*adj OR (95% CI)*
**CC**	54 (9.4%)	97 (9.9%)	***0.0343***	*0.87 (0.55–1.35) [p value: 0.53]*
**CG**	264 (45.8%)	382 (39.1%)		***1.33 (1.03–1.72) [p value: 0.029]***
**GG**	259 (44.9%)	498 (51.0%)		*ref.*
**ALLELES**				
**C (MAF)**	372 (32.2%)	576 (29.5%)	*0.11*	*1.07 (0.89–1.29) [p value: 0.47]*
**G**	782 (67.8%)	1378 (70.5%)		*ref.*
**HWE (*p* value)**	0.26	0.0625		

Statistically significant values are written in bold. Abbreviations: HWE—Hardy–Weinberg equilibrium; MAF—minor allele frequency.

**Table 3:** Association between rs7069102 and DR in T2DM patients according to dominant and recessive models of inheritance.

SIRT1 rs7069102	Case/Patients Without DR	*p Value*	*adj OR (95% CI)*
**DOMINANT**		***0.0204***	
**CC + CG**	318 (55.1 %)/479 (49.0%)		***1.30 (1.02–1.65) [p value: 0.036]***
**GG**	259 (44.9%)/498 (51.0%)		*ref.*
**RECESSIVE**			
**CC**	54 (9.4%)/97 (9.9%)	*0.71*	*0.75 (0.49–1.15) [p value: 0.19]*
**CG + GG**	523 (90.6%)/880 (90.1%)		*ref.*

Statistically significant values are written in bold. Adjusted for: BMI, waist circumference, fasting glucose, T2DM duration, HbA1c (%), CAD, D. neuropathy, insulin therapy.

**Table 4:** Distribution of genotypes and alleles of rs1800629 and logistic regression.

**TNF-α rs1800629**	**Case** **(N = 577)**	**Patients Without DR** **(N = 977)**	***p Value***	***adj OR (95% CI)***
**AA**	23 (4.0%)	24 (2.5%)	*0.15*	*1.44 (0.72–2.87) [p value: 0.30]*
**AG**	155 (26.9%)	245 (25.1%)		*1.22 (0.92–1.62) [p value: 0.16]*
**GG**	399 (69.2%)	708 (72.5%)		*ref.*
**ALLELES**				
**A (MAF)**	201 (17.4%)	293 (15.0%)	*0.07*	*1.21 (0.96–1.53) [p value: 0.10]*
**G**	953 (82.6%)	1661 (85.0%)		*ref.*
**HWE (*p* value)**	0.11	0.61		
**DOMINANT**				
**AA + AG**	178 (30.8%)	269 (27.5%)	*0.16*	*1.24 (0.95–1.62) [p value: 0.11]*
**GG**	399 (69.2%)	708 (72.5%)		*ref.*
**RECESSIVE**				
**AA**	23 (4.0%)	24 (2.5%)	*0.09*	*1.37 (0.68–2.7) [p value: 0.37]*
**AG + GG**	554 (96.0%)	953 (97.5%)		*ref.*

Adjusted for: BMI, waist circumference, fasting Glucose, T2DM duration, CAD, DN, and insulin therapy.

**Table 5:** Analysis of SIRT1 rs7069102 genotypes with waist circumference, fasting glucose and HbA1c levels.

Cases	*p* Value
Waist circumference	0.106
Fasting glucose	0.289
HbA1c	0.579
**Patients Without DR**	
Waist circumference	0.435
Fasting glucose	0.523
HbA1c	0.174

## Data Availability

The data presented in this study are available on request from the corresponding author due to sensitive information (patients’ clinical data).

## Abbreviations

The following abbreviations are used in this manuscript:

T2DM	Type 2 diabetes mellitus
SIRT1	Sirtuin 1
DR	Diabetic retinopathy
TNF-α	Tumor necrosis factor α
DN	Diabetic nephropathy
MMP9	Matrix metalloproteinase 9
BMI	Body mass index
SBP	Systolic blood pressure
DBP	Diastolic blood pressure
HDL	High-density lipoprotein
LDL	Low-density lipoprotein
TGS	Triglycerides
AH	Arterial hypertension
CAD	Cardiovascular disease
MENA	Middle East and North Africa
